# ADAR1 is a prognostic biomarker and is correlated with immune infiltration in lung adenocarcinoma

**DOI:** 10.1002/cam4.6044

**Published:** 2023-05-10

**Authors:** Wendi Yang, Kehong Chen, Qian Yu, Rongxin Liao, Hengqiu He, Yuan Peng, Zhenzhou Yang, Xiaoyue Zhang

**Affiliations:** ^1^ Department of Cancer Center The Second Affiliated Hospital of Chongqing Medical University Chongqing China

**Keywords:** adenosine deaminase 1, lung adenocarcinoma, lymph node metastasis, tumor microenvironment

## Abstract

**Background:**

Lung adenocarcinoma (LUAD) is a common subtype of non‐small cell lung cancer with high morbidity and mortality rates and is usually detected at advanced stages because of the early onset of metastasis. Adenosine deaminase RNA‐specific 1 (ADAR1) is an RNA editing enzyme that catalyzes the important physiological process of adenosine‐to‐inosine editing and has been shown to participate in the progression of LUAD. Increasing evidence has suggested that immune infiltration of the tumor immune microenvironment has prognostic value for most human solid organ malignancies; however, much is unknown about the functions of ADAR1.

**Methods:**

The expression of ADAR1 was analyzed in The Cancer Genome Atlas ‐LUAD database and validated in our LUAD cohort. To assess the prognostic value of ADAR1, Kaplan‐Meier survival analyses and Cox regression analyses were carried out in LUAD cohorts. The association between ADAR1 and LUAD immune infiltrates via analyses of cell‐type identification by estimating relative subsets of known RNA transcripts. Furthermore, multiplex immunohistochemistry was used to confirm the relationship between ADAR1 expression and immune cells in the present cohort of patients with LUAD.

**Results:**

ADAR1 was highly expressed in LUAD tissues and closely correlated with lymph node metastasis (LNM) (*p* < 0.01), advanced tumor stage (*p* < 0.05), and poor patient prognosis (*p* < 0.01), thus indicating that increased ADAR1 contributed to the progression of LUAD. LUAD with high ADAR1 expression can metastasize to lymph nodes that express more ADAR1 than the primary lesion. In addition, M0 macrophages and M2 macrophages increased and CD4^+^T cells decreased in LUAD tissues with high ADAR1 expression. And the expression of ADAR1 in lymph node metastases was negatively correlated with the contents of CD4^+^T cells (*p* = 0.0017) and M1 macrophages (*p* = 0.0037).

**Conclusion:**

The findings of our study suggested that ADAR1 may be useful in predicting prognosis and LNM in LUAD, and may serve as a promising immune‐related molecular target for LUAD patients.

## 1 INTRODUCTION

Lung adenocarcinoma (LUAD), which is the most common subtype of non‐small cell lung cancer (NSCLC), exhibits marked morphological heterogeneity and is composed of tumor cells of multiple histological subtypes.^1^ The overall 5‐year survival rate of LUAD is less than 15% due to rapid invasion and metastasis at the early stage of the disease.^2^ When lymph node metastasis (LNM) occurs, cancer patients often have a worse survival rate (even those patients with early diagnosis of the disease).^3^ In recent years, although considerable progress has been made in the clinical treatment of LUAD, the benefits for patients are still small. Therefore, the exploration of new prognostic markers and therapeutic targets in LUAD is of great clinical significance.

Adenosine deaminase RNA specific 1 (which is the first identified and most widely expressed isoform of the ADAR family) catalyzes adenosine‐to‐inosine RNA editing in the double‐stranded RNA reverse repeat element,^4^ which is a dynamic modification that can produce a very diverse transcriptome in a combinatorial manner.^5^ ADAR1‐mediated RNA editing is essential for various biological processes, and its dysregulation leads to aberrant editing of its substrates, which may contribute to cancer development and progression.^6^ Previous studies have shown that the aberrant expression of ADAR1 participates in the development of breast cancer, esophageal cancer, multiple myeloma, and other cancers.^7^, ^8^, ^9^, ^10^, ^11^ And loss of ADAR1 function can enhance the tumor response to PD‐1 blockade and improve the tolerance of tumor immunotherapy.^12^ In addition, ADAR1 promotes the malignant progression of NSCLC, yet its effect on LUAD remains to be elucidated.^13^ Therefore, we focused on the role played by ADAR1 in the tumorigenesis, evolution, and metastasis of LUAD.

The initiation and development of tumors cannot be separated from the tumor immune microenvironment (TME), which is a complex and dynamic collection of tumor cells, stromal cells, cancer‐associated fibroblasts, endothelial cells, immune cells, extracellular matrix, chemokines, and cytokines.^14^ TME provides malignant cells with physical support, sufficient nutrients, and oxygen and helps with the establishment of an immunosuppressive environment and immune evasion, thus facilitating the growth, invasion, and metastasis of tumors.^15^ Among the various functionally different cells in the TME, immune cells (both resident and infiltrated) play a vital role in tumor development and progression, which can be a double‐edged sword for tumors or therapy.^16^, ^17^ Immune cells could form a kind of immunosurveillance composed of various proinflammatory cells, such as M1 macrophages and CD8^+^ cytotoxic T cells, although they are rapidly exhausted.^18^, ^19^ In contrast, many immunosuppressive cells, such as M2 macrophages, myeloid‐derived suppressor cells (MDSCs), and CD4^+^ type 2 helper cells, make up a tumor‐promoting TME.^20^, ^21^, ^22^ Several studies demonstrate that ADAR1 participates in the immune recognition and immune escape of tumor cells.^6^, ^23^ However, no relevant reports have investigated the role of ADAR1 in the construction of the immune microenvironment of LUAD tissues. Therefore, the evaluation of whether ADAR1 affects the progression and metastasis of LUAD through the TME is valuable for further improving the clinical management of this disease.

To this end, in this study, we combined the analysis of public cancer databases and our clinical cohort to explore the potential role of ADAR1 in LUAD and performed a tumor‐immune interaction study. The results would provide new immunological insights into the underlying mechanisms of LUAD progression and metastasis.

## 2 MATERIALS AND METHODS

### 2.1 Analysis of data from TCGA

The transcriptomic expression and clinical data of 998 NSCLC patients (495 lung squamous carcinoma [LUSC] and 503 LUAD) were downloaded from The Cancer Genome Atlas (TCGA) database (https://tcga‐data.nci.nih.gov).^24^ For survival analysis, all LUSC and LUAD patients were divided into two groups (high expression and low expression) according to the mean value of specific genes in the samples, which were then modeled by survival package and visualization. Differential expression analysis and mapping were performed using the R package Limma (version 3.42.2),^25^ and the differentially expressed genes (DEGs) were divided into high expression group and low expression group based on the median of ADAR1 expression in TCGA‐LUAD. Hazard ratios (HRs) with 95% confidence intervals (CIs) and log‐rank *p*‐values were calculated.

### 2.2 Identification of gene ontology (GO) category

GO analysis includes three categories: molecular function, biological process, and cellular component.^26^ We performed GO annotation for DEGs using the clusterProfiler (version 3.10.1) package.^27^ The adjusted *p*‐value < 0.05 was set as the cut‐off criterion. The connections between the most significant GO terms and participating genes were visualized by GOenrich package with a network diagram.

### 2.3 KEGG pathway analysis

Kyoto Encyclopedia of Genes and Genomes (KEGG) consists of graphical diagrams of biochemical pathways, including metabolic pathways and some known regulatory pathways.^28^ We use the clusterProfiler (version 3.10.1) package to perform KEGG pathway analysis of DEGs to reveal the biochemical pathways they are involved in. *p* < 0.05 and adjusted *p* < 0.05 were set as the threshold values.

### 2.4 Patient recruitment and sample collection

Samples from a total of 100 patients with LUAD and 48 patients with LUSC who underwent surgical resection in the Second Affiliated Hospital of Chongqing Medical University from December 2017 to September 2019 were collected, including cancer tissues and adjacent normal tissues. In addition, 16 lymph node tissues with LUAD metastasis were also collected from the Second Affiliated Hospital of Chongqing Medical University between 2018 and 2021. The specimens were fixed with 4% neutral paraformaldehyde, embedded in paraffin, sectioned, and histopathologically diagnosed by a senior pathologist using HE staining. Then, these LUAD tissues and adjacent normal tissues with clinicopathological features and follow‐up information were used to make two tissue microarray wax blocks using a tissue microarrayer (Leica). At the same time, 16 pairs of primary LUAD tissues and their corresponding lymph node metastases were collected. The tissues were fixed with 4% paraformaldehyde and embedded in paraffin to make tissue sections with a thickness of 4 μm. This study was reviewed and approved by the Ethics Committee of the Second Affiliated Hospital of Chongqing Medical University. Written informed consent from all patients was obtained.

### 2.5 Immunohistochemistry (IHC)

IHC staining was performed using a kit purchased from ZSGB‐Bio (ZLI‐9019). Specifically, paraffin sections were deparaffinized in fresh xylene and then sequentially placed in gradient ethanol (95%–85%–75%) for rehydration. Retrieval of antigen was then conducted by heating in sodium citrate solution (10 mM, pH 6.0) for 15 min in a microwave oven. Next, endogenous peroxidase was blocked by incubation with endogenous peroxidase blocking agent for 10 min at room temperature. After that, the sections were incubated with 100 μL of anti‐ADAR1 primary antibody (1:100, #81284, Cell Signaling Technology) overnight at 4°C. Finally, after 20 min of incubation at 37°C with secondary antibodies conjugated with horseradish peroxidase, development was achieved using freshly prepared diaminobenzidine solution for 5 min. Images were acquired on an Evos Fl Color Imaging System (Thermo Fisher Scientific). Five random fields were selected for each section and averaged. Quantification of staining intensity was performed using ImageJ 1.50i software. To assess ADAR1 expression levels, we used a graded semiquantitative scoring system. We performed a semi‐quantitative analysis based on the intensity of cell staining and the positive expression of the ADAR1 protein. The positive expression of ADAR1 protein was localized in the nucleus, and the positive result was brown yellow, or tan. Staining intensity was scored as 0 (no cell staining), 1 (light brown particles), 2 (brown yellow particles), and 3 (tan particles). The percentage of positive cells was scored as 0 (≤5%), 1 (5%–25%), 2 (26%–50%), 3 (51%–75%), and 4 (≥75%). The product of these two is the immunoreactivity score (IRS): 0 (negative), 1 and 2 (weak staining), 3 and 4 (moderate staining), 6 and 8 (moderately severe staining), and 9 and 12 (strong staining). ADAR1 high‐expression or low‐expression refers to IRS scores of 7–12 or 0–6, respectively.

### 2.6 Multiplex immunohistochemistry staining (mIHC)

mIHC was implemented using a PANO Multiplex IHC kit (5‐color) (#10080100100, Panovue, China) according to the manufacturer's instructions. After sequential application of ADAR1 (#81284, Cell Signaling Technology), CD4 (ab183685, Abcam), CD68 (#97778, Cell Signaling Technology) and CD8 (ab237709, Abcam) antibodies or CD20 (#48750, Cell Signaling Technology), CD84 (ab131256, Abcam), CD86 (#91882, Cell Signaling Technology), and CD206 (#24595, Cell Signaling Technology) antibodies to the sections, horseradish peroxidase‐conjugated secondary antibody incubation and tyramide signal amplification which were heated by microwave each time were conducted. Then DAPI was used to stain the nuclei. Pictures were captured using the Mantra System (PerkinElmer), and five random fields were selected for each slide.

### 2.7 Tumor‐infiltrating immune cell assessment

In order to explore the effect of ADAR1 on the TME, cell‐type identification by estimating relative subsets of known RNA transcripts (CIBERSORT; http://cibersort.stanford.edu/) was used to evaluate the proportion of 22 types of immune cells infiltrated in LUAD. It is a versatile computational method for quantifying cell fractions from bulk tissue gene expression profiles, which is a useful approach for high throughput characterization of diverse cell types, such as TILs, from complex tissues.^29^ Furthermore, in benchmarking experiments, CIBERSORT was more accurate than other methods in resolving closely related cell subsets and in mixtures with unknown cell types (e.g., solid tissues).^30^

### 2.8 Statistical analysis

SPSS software (version 23.0; IBM) or GraphPad Prism 7 was used for statistical analysis of the data, and ImageJ (National Institutes of Health) was used to analyze the immunohistochemical staining results and process images. The clinicopathological characteristic data are expressed as the mean ± standard deviations (SD), the *t*‐test was used to compare the mean between two groups, and the *χ* ^2^ test was used for univariate analysis. A Cox regression analysis model was fitted. Univariate and multivariate Cox proportional hazards regression analyses were performed to determine the clinicopathological parameters of overall survival (OS), and the results were expressed as HR and 95% CI. IHC and mIHC results were analyzed utilizing unpaired *t*‐tests and are presented as the means ± SD or as scatter plots. Data containing more than two groups were analyzed by ANOVA. For all the above analyses, a *p‐*value less than 0.05 or 0.01 was considered statistically significant.

## 3 RESULTS

### 3.1 ADAR1 is upregulated in LUAD and associated with poor OS

**FIGURE 1:**
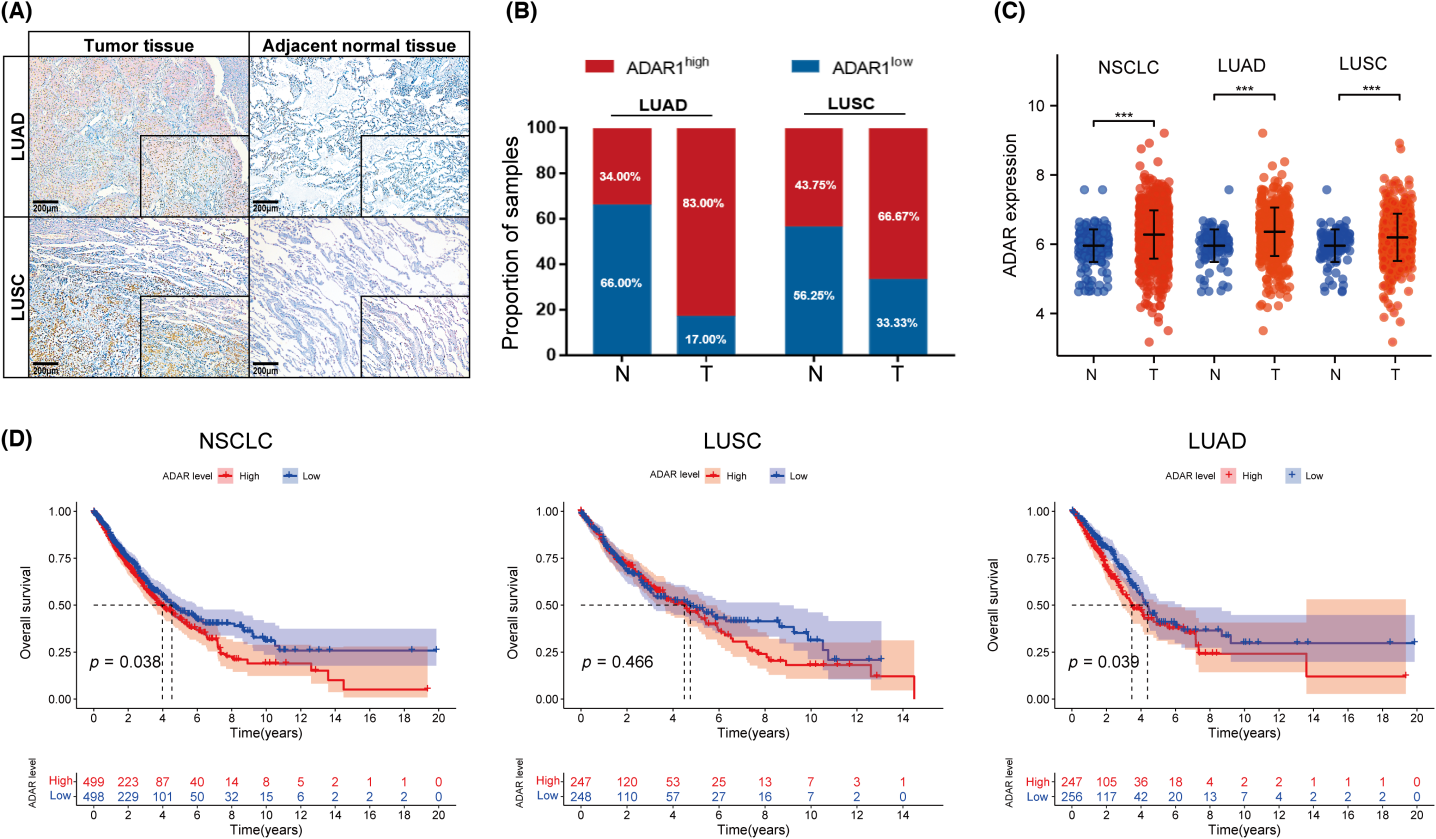
Excessive expression of adenosine deaminase RNA‐specific 1 (ADAR1) contributes to poor prognosis in non‐small cell lung cancer (NSCLC). (A) Representative images of IHC staining with an ADAR1 antibody on lung adenocarcinoma (LUAD) and lung squamous carcinoma (LUSC) tumor tissues and their adjacent normal lung tissues. Scale bars: 200 μm. (B) Proportion of ADAR1^high^ and ADAR1^low^ cases of LUAD or LUSC. (C) ADAR1 mRNA expression levels in NSCLC, LUAD, and LUSC (***p < 0.001). (D) Overall survival curves based on ADAR1 mRNA expression in patients with NSCLC, LUSC, or LUAD.

To detect the expression level of ADAR1 in NSCLC patients, we collected 100 LUAD tissues and 48 LUSC tissues (with identical amounts of corresponding normal adjacent tissues) from The Second Affiliated Hospital of Chongqing Medical University. The results from both LUAD and LUSC patients showed that ADAR1 was much more highly expressed in the tumor than in the normal adjacent tissues (Figure 1A). Based on the intensity and patterns of IHC staining, we defined ADAR1^high^ and ADAR1^low^ samples to represent high‐expression and low‐expression of ADAR1 in those related patients, respectively. We found that 83.0% of LUAD patients and 66.67% of LUSC patients manifested as ADAR1^high^ (83 cases and 32 cases, respectively), which indicated that most of the NSCLC patients had higher expression of ADAR1 (Figure 1B). Consistent with these findings, the patients with LUAD and LUSC with their matched normal adjacent tissues from TCGA RNA‐sequencing data also demonstrated that the expression of ADAR1 was upregulated in NSCLC patients (Figure 1C). Afterward, we explored the relationship between ADAR1 and the OS of NSCLC patients. According to Kaplan–Meier survival analysis, NSCLC patients with high ADAR1 expression had a poorer OS than those with low ADAR1 expression. After differentiating LUAD patients from LUSC patients for analysis, we found that ADAR1 did not affect the OS of the LUSC patients, but affected the OS of the LUAD patients. Specifically, LUAD patients with high ADAR1 expression had a poorer OS than those with low ADAR1 expression, which is a result similar to that of NSCLC patients (Figure 1D). These results indicated that elevated ADAR1 in NSCLC patients is accompanied by a worse prognosis, especially in LUAD patients.

### 3.2 ADAR1 is associated with advanced pathological stage and unfavorable prognosis in LUAD

**FIGURE 2:**
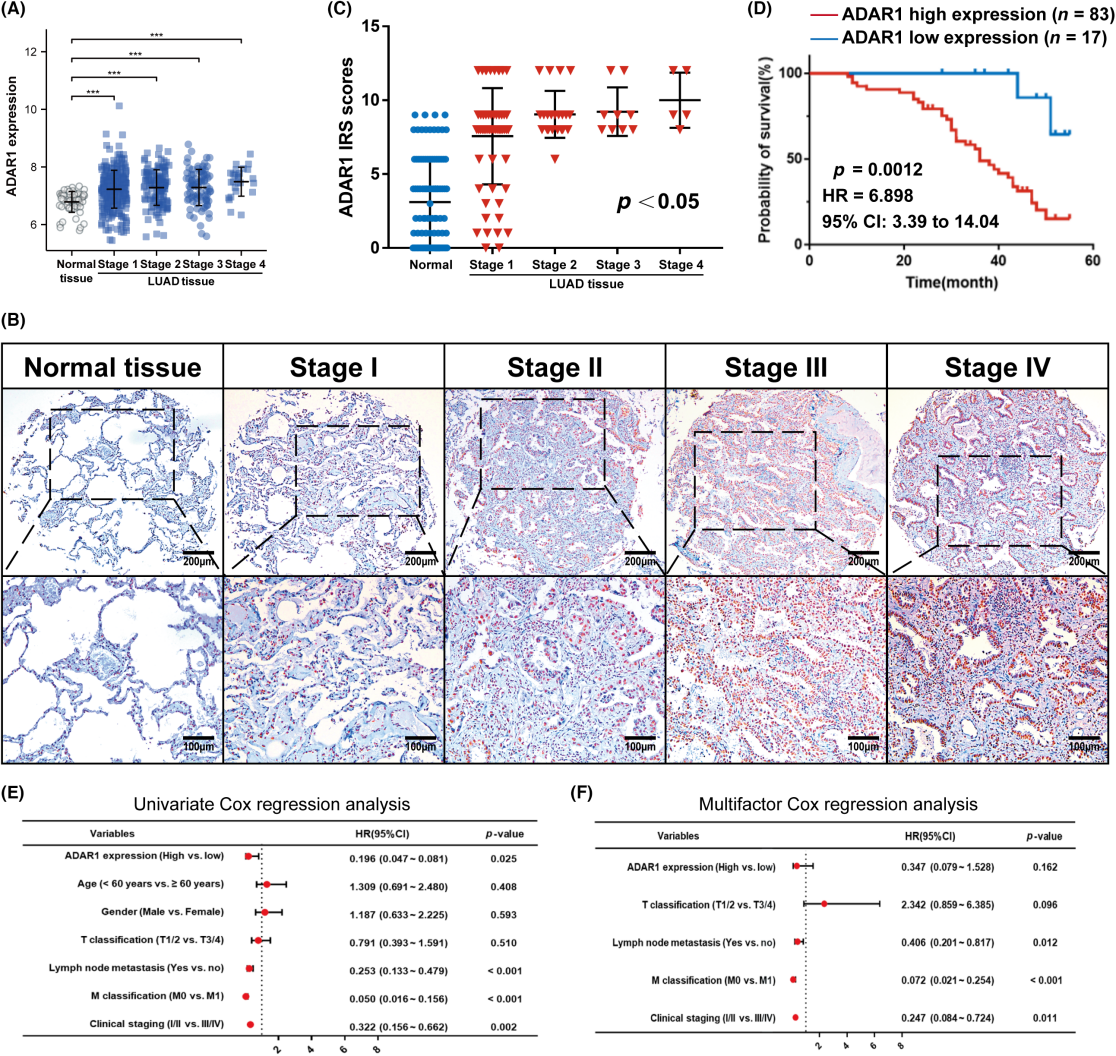
Adenosine deaminase RNA‐specific 1 (ADAR1) expression is associated with the clinicopathological characteristics and prognosis of patients with lung adenocarcinoma (LUAD). (A) Analysis of ADAR1 expression levels based on the clinical stages of The Cancer Genome Atlas‐LUAD. (B) The expression levels of ADAR1 in stage I, stage II, stage III, stage IV, and normal tissues of patients with LUAD from the present cohort (*n* = 100). (C) Immunoreactivity score of ADAR1 expression in patients with LUAD at different stages. (D) K‐M survival analysis of overall survival for LUAD patients according to the level of ADAR1 protein expression. (E) Univariate and (F) multivariate Cox analyses of ADAR1 in LUAD patients. (***p < 0.001, **p < 0.01, ns *p* > 0.5 nonsignificant).

It is well established that the tumor node metastasis classification and clinical staging reflect the severity and malignancy of cancers and are important in assessing the prognosis of patient survival.^31^ Due to the fact that ADAR1 was more closely related to the OS of LUAD patients (Figure 1D), we mainly focused on the role of ADAR1 in the pathological stage and prognosis of LUAD patients. The results from the TCGA database suggested that LUAD tissues expressed more ADAR1 than normal lung tissues at different clinical stages (Figure 2A). To test this finding, we performed IHC of ADAR1 in two tissue chips containing 100 LUAD specimens and their adjacent normal lung tissues. We found that the ADAR1 protein positivity rate was remarkably higher in LUAD tissues (83.0% vs. 17.0%) than in normal tissues (34.0% vs. 66.0%; Figure 1C). After analyzing the correlation of ADAR1 expression level with age, gender, LNM, T stage, N stage, M stage, and clinical stage, we found that ADAR1 was mainly related to LNM, N stage, and clinical stage (Table S1, Figure 2B,C); in addition, patients with a more advanced clinical stage tended to express more ADAR1 (Figure 2B,C).

To further explore the clinical significance of ADAR1 in LUAD patients, we first compared the OS of the 100 patients collected and the disease‐free survival (DFS) of the 55 patients who could be traced back to the time of surgery via Kaplan–Meier survival analysis. The results both showed that the survival time of patients with high ADAR1 expression was significantly shorter than that of patients with low expression (*p* < 0.05; Figure 2D; Figure S1). In the results of clinicopathologic feature analysis, we found that ADAR1 was closely correlated with LNM (*p* = 0.002) and advanced tumor stage (*p* = 0.042; Table S1). Moreover, in patients without LNM or with clinical stage I and II, the survival curve distribution of ADAR1 high expression group was significantly different from that of low expression group (*p* = 0.0239, *p* = 0.0065; Figure S2). We subsequently verified the predictive potential of ADAR1 for the OS of LUAD patients by using univariate and multivariate Cox regression analyses. The univariate analysis indicated that clinical stage (HR = 0.322, *p* = 0.002), M classification (HR = 0.050, *p* < 0.001), LNM (HR = 0.253, *p* < 0.001), and ADAR1 expression (HR = 0.196, *p* = 0.025) contributed to poor OS in patients with LUAD (Figure 2E). In the multivariate analysis, clinical stage (HR = 0.247, *p* = 0.01), M classification (HR = 0.072, *p* < 0.001), and LNM (HR = 0.406, *p* = 0.012) were independent prognostic factors for LUAD patients (Figure 2F). Therefore, ADAR1 is of great importance in predicting the prognosis of LUAD patients. More importantly, the high expression of ADAR1 can be used as a poor prognostic factor for LUAD patients without LNM or with clinical stage I and II.

### 3.3 LUAD with high ADAR1 expression has a tendency toward lymph node metastasis

**FIGURE 3:**
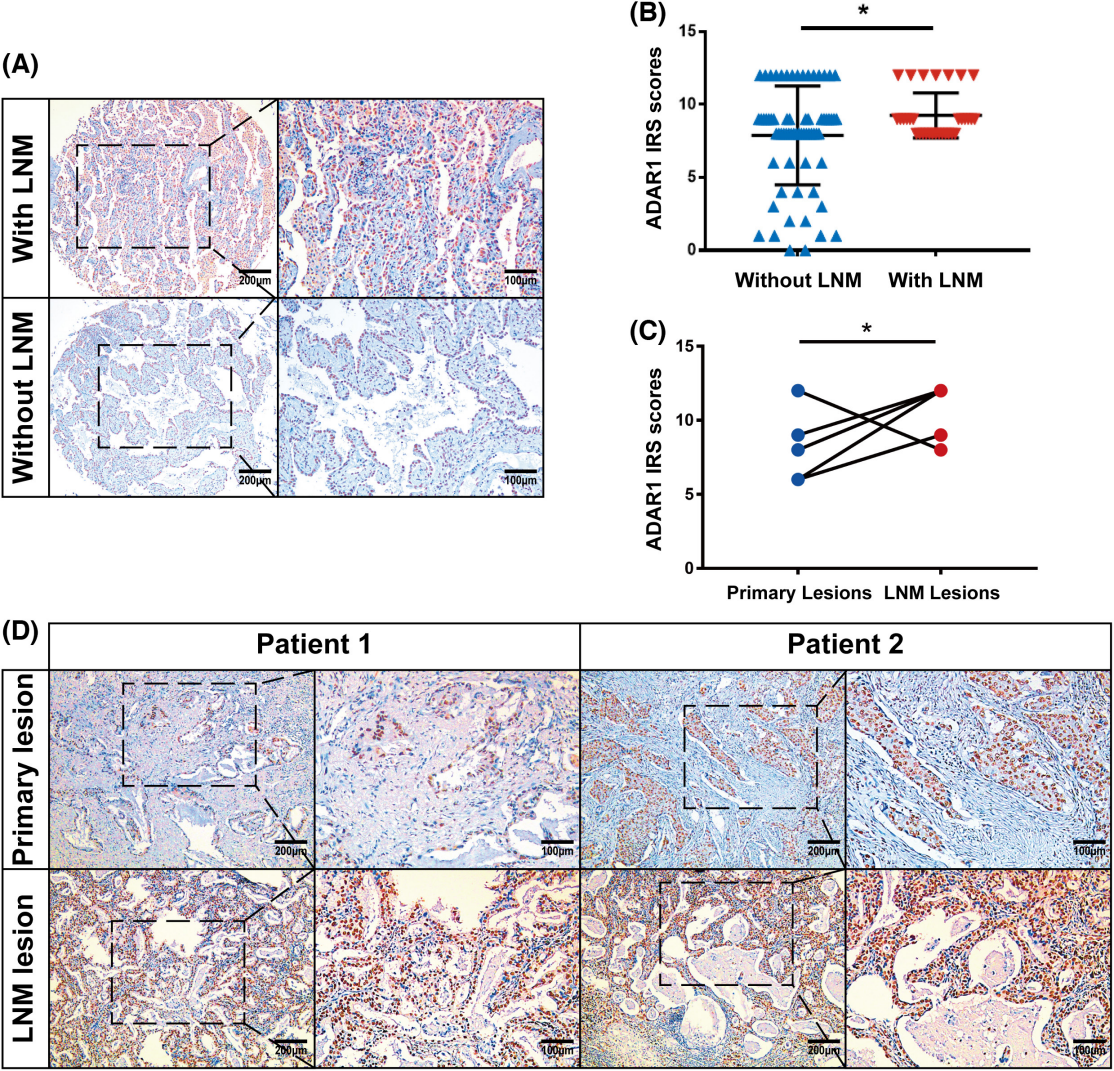
Elevated adenosine deaminase RNA‐specific 1 (ADAR1) expression is associated with lymph node metastasis (LNM) in lung adenocarcinoma (LUAD). ADAR1 expression level in LUAD with or without LNM (A) and their immunoreactive scores (B). Scale bars: 100 μm. ADAR1 expression level in primary LUAD or paired LNM lesions (C) and their immunoreactive scores (D). (mean ± SD, Student's *t*‐test, **p < 0.01, *p < 0.05).

Metastasis is the leading cause of cancer‐related death. For many solid malignancies, lymph node involvement is a precursor to distant metastatic disease, and the latest studies suggest that LNM can promote distant metastases by inducing tumor immune tolerance,^32^ which is inextricably linked to the poor prognosis of patients. In this study, we noted that ADAR1 expression was strongly associated with LNM in the collected 100 LUAD samples (Table S1). We then examined the protein level of ADAR1 in the primary lesions of LUAD patients with or without LNM in our cohort and found that ADAR1 was significantly increased in patients with LNM (Figure 3A). Moreover, IRS analysis further demonstrated a significant difference in IHC scores between the two groups (Figure 3B). Additionally, we also estimated ADAR1 expression levels in 16 LNM tissues of LUAD patients in our cohort. As shown in Figure 3D, ADAR1 expression levels in LNM were significantly higher than those in paired primary lesions, and the IRS results also showed that LNM scored higher than their matched primary lesions in 16 paired specimens (Figure 3C). Altogether, these results demonstrated the potential role of ADAR1 in the development of LNM in LUAD.

### 3.4 ADAR1 correlates with the tumor‐promoting TME in LUAD

**FIGURE 4:**
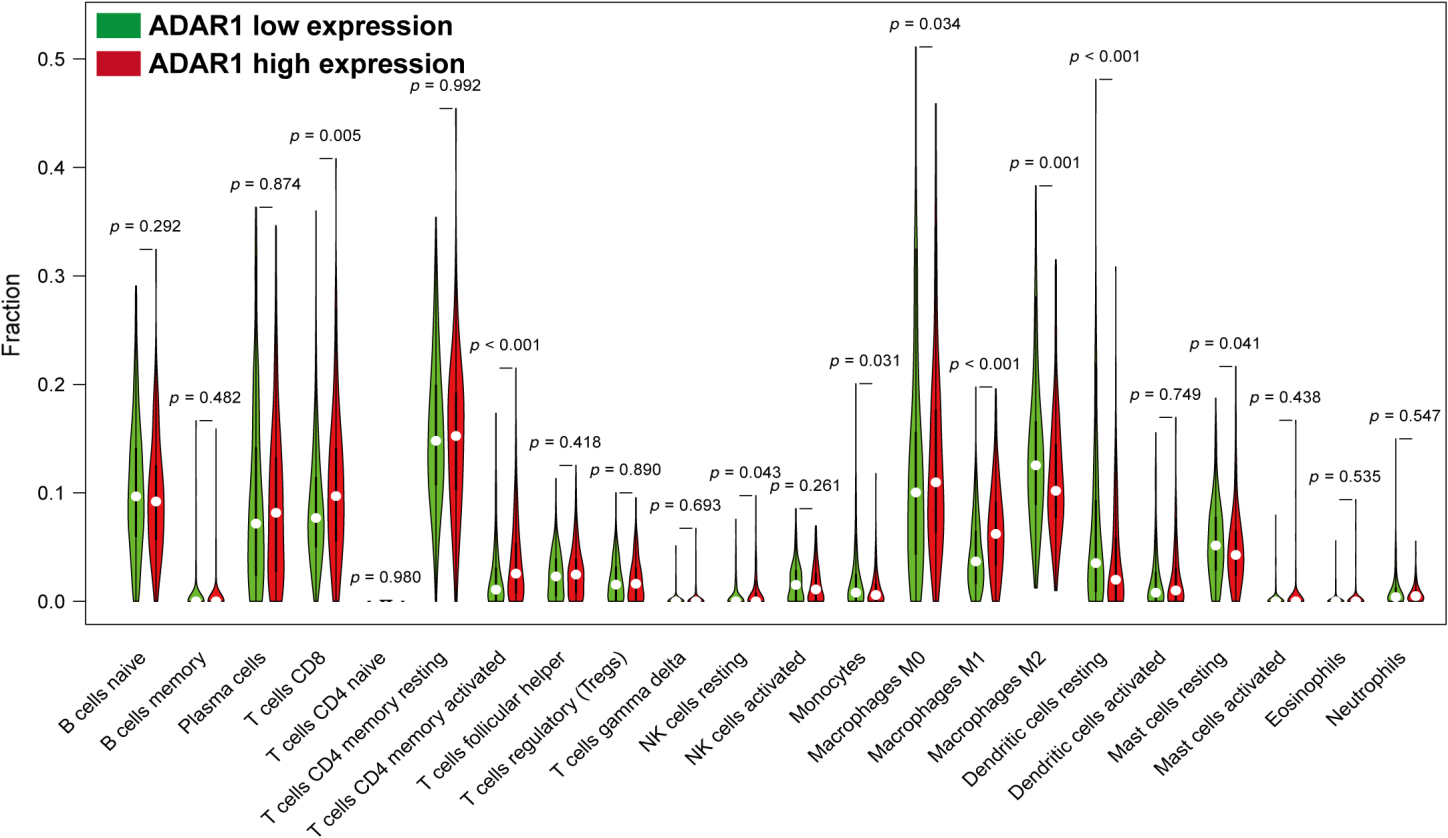
Correlation of adenosine deaminase RNA‐specific 1 (ADAR1) with tumor immune infiltration in lung adenocarcinoma (LUAD). Correlation between ADAR1 and 22 tumor‐infiltrating immune cells in LUAD samples as analyzed via cell‐type identification by estimating relative subsets of known RNA transcripts. An absolute value of |Rho| > 0.1 and *p* < 0.05 implied that the ADAR1 expression level was significantly associated with the number of immune cells.

The tumor microenvironment is considered one of the most important factors affecting the prognosis of NSCLC patients. The degree and proportion of immune cell infiltration have different effects on tumor prognosis.^33^, ^34^ Therefore, we assessed whether (or to what extent) ADAR1 alters the distribution of immune cells within the local TME of LUAD. By using CIBERSORT, we found that there were significant differences in the infiltration levels of a considerable number of immune cells between the ADAR1 high and low expression groups, which undoubtedly indicated that ADAR1 is likely to be a key regulator in the TME. Specifically, CIBERSORT demonstrated that the ADAR1 high expression group had a higher proportion of CD8^+^ T cells, activated CD4^+^ memory T cells, resting natural killer cells, M0 macrophages, and M1 macrophages in tumor tissues, whereas there were more monocytes, resting dendritic cells, M2 macrophages and resting mast cells infiltrating in the low expression group (Figure 4).

**FIGURE 5:**
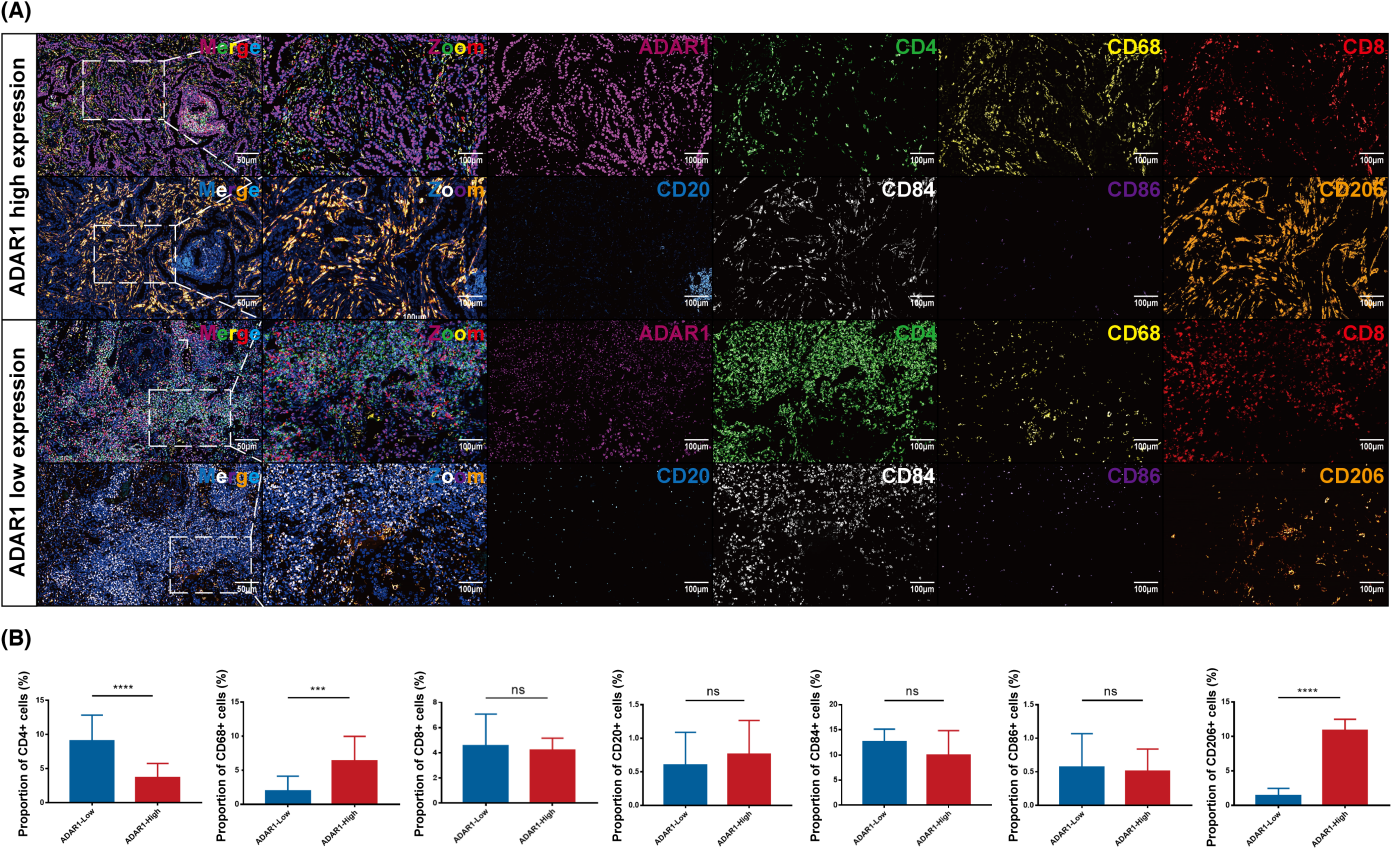
Adenosine deaminase RNA‐specific 1 (ADAR1) is involved in immune cell infiltration in lung adenocarcinoma (LUAD) tissue. (A) Multiplex immunohistochemistry staining of LUAD sections of the high‐ (upper panel) or low‐ (lower panel) ADAR1 group. (B) Histograms displaying the quantitative data of CD4^+^, CD68^+^, CD20^+^, CD84^+^, CD86^+^, and CD206^+^ cells in the ADAR1‐high and ADAR1‐low groups. ADAR1^+^ (magenta), CD4^+^ T cells (CD4^+^, green), pan‐macrophages (CD68^+^, yellow), CD8^+^ T cells (CD8^+^, red), B cells (CD20^+^, celeste), myeloid‐derived suppressor cells (CD84^+^, white), M1 macrophages (CD86^+^, purple), and M2 macrophages (CD206^+^, orange). (mean ± SD, Student's *t*‐test, ^****^ *p* < 0.0001, ***p < 0.001, **p < 0.01, *p < 0.05, ns *p* > 0.05, not significant).

We next determined some markers of immune cells on our specimens at the protein level to further explore the association between ADAR1 and those infiltrating cells. Multiplex IHC, which is a well‐established method to characterize tumor immune infiltration and to evaluate tumor‐immune interactions, was employed in 16 LUAD patients' tissues.^35^ We observed that CD4^+^ T cells exhibited less infiltration in tumor tissues with high ADAR1 expression. The number of infiltrating CD8^+^ T cells was not significantly different between the high ADAR1 group and the low ADAR1 group in LUAD. (Figure 5). CD84 is a specific marker of MDSCs that is highly expressed in primary LUAD, thus indicating the immunosuppressive tumor microenvironment of tumor tissue. However, there was no significant correlation between CD84 and ADAR1 (Figure 5). Moreover, tumor‐associated macrophages are functionally heterogeneous and mainly divided into two subtypes: classically activated M1 macrophages (proinflammatory macrophages) and alternatively activated M2 macrophages (anti‐inflammatory macrophages). M1 macrophages express CD86, whereas M2 macrophages are characterized by increased CD206 expression. Consequently, mIHC showed that LUAD tissues with high ADAR1 expression had more M0 and M2 macrophages infiltration (Figure 5). Meanwhile, we performed mIHC on histological sections of 16 LUAD LNM. The results showed that high ADAR1 expression appeared to be infiltrated by fewer CD4^+^ T cells and more M2 macrophages (Figure S3).

Furthermore, in order to explore the biological processes and signaling pathways involved in ADAR1 in LUAD, we divided the DEGs into high expression group and low expression group based on the median of ADAR1 expression in TCGA‐LUAD and then screened out the DEGs for GO and KEGG enrichment analysis. Among the 8139 up‐regulated genes, we found that the top 10 GO annotations were mainly biological processes and cellular components, such as wound healing, cilium movement, extracellular structure organization, and extracellular matrix organization. And KEGG‐enriched signaling pathways included pancreatic secretion, salivary secretion, drug metabolism‐cytochrome P450, and other metabolic secretion‐related pathways (Figure S4). In addition, we noted that genes in the PPAR signaling pathway were also upregulated when ADAR1 was overexpressed. PPAR pathway has been confirmed to be closely related to lung cancer, breast cancer, colon tumors, and even brain metastases.^36^, ^37^, ^38^, ^39^ Therefore, we hypothesize that there is also some potential link between ADAR1, which is highly expressed in LUAD, and PPAR pathway, which in turn promotes the malignant progression of LUAD and the formation of a suppressive immune microenvironment.

Thus, ADAR1 may play an important role in LUAD. Taken together, ADAR1 may participate in building the TME, especially driving the formation of an immunosuppressive TME in LUAD and further promoting tumor cell metastasis.

## 4 DISCUSSION

Although the prognosis of LUAD has improved due to many advances in diagnosis and treatment over the past few decades, LUAD remains one of the current leading causes of cancer death, and the search for new prognostic biomarkers remains urgent. Modifications at the transcriptional level can promote the transformation of normal cells into malignant tumors and can help tumor cells evade immune recognition.^6^ ADAR1 editing and its aberrant expression leads to the development of malignant tumors, thus we explored its role in LUAD. Herein, we present a comprehensive analysis of both publicly accessible databases and independent external cohorts regarding the relationship between ADAR1 and LUAD. The results showed that ADAR1 was overexpressed at both the mRNA and protein levels in NSCLC. Notably, this phenomenon is more prominent in LUAD, wherein high ADAR1 expression suggests worse prognosis and OS, thus indicating that ADAR1 may be a tumor‐promoting gene involved in LUAD progression.

Due to the fact that many solid tumors that colonize lymph nodes are susceptible to the induction of immune tolerance, such tumors are more likely to spread to other distant organs.^32^ Indeed, LUAD is most likely to metastasize to lymph nodes. We noted that high ADAR1 expression was strongly associated with LNM and the clinical stage of LUAD in our clinical cohort. Moreover, the Cox multivariate regression analysis confirmed that LNM was an independent prognostic factor for LUAD. Therefore, we investigated the role of ADAR1 in LNM and found that LUAD patients with high ADAR1 expression had an increased risk of LNM. In addition, regardless of the ADAR1 expression level in LUAD primary lesions, the corresponding lymph node metastases had higher ADAR1 expression levels. ADAR1 has been shown to be important for the suppression of innate immunity. In previous studies, ADAR1‐mediated RNA editing was found to be associated with melanoma growth and metastasis.^7^ Shen et al. also reported that the proliferation and metastasis of pancreatic ductal carcinoma are regulated by ADAR1 through a negative feedback loop.^40^ These findings are consistent with our current results and support the critical role of ADAR1 in the invasion and metastasis of LUAD. Tumor metastasis is a complex process, that largely depends on the interaction between cancer cells and the tumor stroma; specifically, malignant tumors induce changes in the stromal environment to promote cell metastasis.^41^ Furthermore, lymph node metastases resist T‐cell‐mediated cytotoxicity, induce antigen‐specific regulatory T cells, and develop tumor‐specific immune tolerance that promotes distant tumor colonization.^42^ Therefore, we hypothesized that ADAR1 could make LUAD tumor cells more likely to metastasize, colonize, and survive by affecting the immune environment of tumor lesions.

The tumor microenvironment containing tumor cells and nontumor cells (such as endothelial cells, immune cells, and fibroblasts) can determine the biological function of tumor cells, which is one of the most important factors affecting the prognosis of LUAD patients.^43^, ^44^ Tumor cells can change their microenvironment and switch immune responses from a tumor‐destructive to a tumor‐promoting mode. Immune cells that are responsible for supporting tumors play a key role in this process.^45^ ADAR1 may drive the transformation of normal cells into malignancies by regulating RNA editing and inhibiting immune responses.^46^, ^47^ Furthermore, ADAR1 expressed by tumor cells may participate in the crosstalk between the TME and tumor, thus contributing to a niche conducive to tumorigenesis and metastasis.^23^, ^47^ Therefore, we analyzed the role of ADAR1 in the immune infiltration of LUAD. The CIBERSORT analysis showed that the distribution patterns of nine immune cells were significantly different between the high and low ADAR1 expression groups. Specifically, compared with the low ADAR1 group, the proportion of CD8^+^ T cells, activated CD4^+^ memory T cells, resting dendritic cells, and M0 and M1 macrophages in the high ADAR1 group was significantly increased, whereas M2 macrophages, monocytes, resting dendritic cells, and mast cells were significantly decreased.

Immune cell infiltration is considered to be a major participant in the process of cancer progression and metastasis.^48^ Different immune cells infiltrating tumors execute various functions, which can be either tumor‐suppressive or tumor‐promoting.^49^ Therefore, the analysis of TILs in primary lesions and metastatic nodules can elucidate the underlying mechanisms and potential treatment strategies.^50^ We inferred from the online database analysis that ADAR1 is critical for the infiltration pattern of immune cells in LUAD. The current results suggest that ADAR1 participates in LUAD LNM. Moreover, the immunosuppressive TME of target organs is indispensable for the colonization and growth of metastatic cancer cells. To investigate what types of immune cells and their relative numbers had infiltrated in LUAD we analyzed the correlation between ADAR1 and immune cell markers via mIHC on serial histological sections from our cohort. The most remarkable result is that CD4^+^ T cells decreased in both primary foci and lymph node metastases, which was contrary to the results from those three online analyses, which indicated an increase in CD4^+^ T cells. However, this phenomenon is in accordance with the fact that tumor development demands an immunosuppressive microenvironment, in which CD4^+^ helper T cells, cytotoxic T cells, and regulatory cells (Tregs) are involved. Although CD4^+^ helper T cells inhibit tumor growth in an indirect fashion by promoting and enhancing the effector functions and memory functions of cytotoxic T lymphocytes, cytotoxic CD4^+^ T cells can directly kill tumor cells in antitumor immunity.^51^ FOXP3‐expressing regulatory T cells suppress the autoimmune response; however, they create an immunosuppressive microenvironment for tumors by inhibiting antitumor effects.^52^ Although Tregs exert the opposite influence to the other two types of CD4^+^ T cells on tumors, the decrease in total CD4^+^ T cells is conceivable during the progression of tumors. More exploration of specific subtypes, quantification, and dynamic variation in CD4^+^ T cells are required to identify the extent and manner by which ADAR1 participates in mediating the LUAD immune response regulated by these cells. Regarding CD8^+^ T cells which are a critical component mediating the killing of tumor cells in the TME,^16^ we observed that there was no significant difference in CD8 signals that were detected in the groups with high and low ADAR1 expression in LUAD lesions, which was not consistent with the results obtained from CIBERSORT. The contradictory expression levels of mRNA and proteins can possibly result from complex posttranslational modifications and translational regulation occurring at the primary locus. Further research is needed to explore these phenomena.

Another crucial category of immune cells in the TME is TAMs. It is widely accepted that M1 macrophages play a significant role in antitumor immunity and mainly regulate proinflammatory processes in the TME. In contrast, M2 macrophages possess pro‐tumor effects such as the promotion of invasion and metastasis.^53^ The CIBERSORT analysis showed that M1 macrophages are enriched in LUAD tissue with high ADAR1 expression and suggest a negative association between ADAR1 and the number of M2 macrophages infiltrating the tumor. In our mIHC findings, more M2 macrophages infiltrated in LUAD primary lesions in the ADAR1 high expression group, and fewer M1 macrophages infiltrated in LNM lesions.

Again, these results implied a suppressive immunological TME in LUAD with high ADAR1 expression. We hypothesize that ADAR1 contributes to an immunosuppressive tumor microenvironment in primary LUAD foci by increasing pro‐cancer M2 macrophages, whereas after metastasis to lymph nodes, ADAR1 contributes to an immunosuppressive microenvironment favorable to tumor cell growth mainly by suppressing anti‐cancer M1 macrophages. Such results may be closely related to the specific function and structure of lymph nodes and the potential role played by ADAR1 in them. As an important immune organ, the lymph node microenvironment is regulated and influenced by multiple systems and processes in the lymph nodes,^54^ and the relationship with immune cells is complex and variable.^55^, ^56^ In addition, the results of our pathway enrichment analysis also demonstrated a potential correlation between ADAR1 promotion of LUAD progression and the PPAR signaling pathway. Each of the three subtypes of PPAR (PPAR‐α, PPAR‐γ, and PPAR‐δ) displays differential tissue distribution and mediates specific functions such as early development, cell proliferation, differentiation, apoptosis, and metabolic homeostasis.^57^, ^58^ Among which PPAR‐γ is known to be expressed in NSCLC cell lines.^59^ The expression level of PPAR‐γ was shown to correlate with malignancy and survival in the lung cancer patient. Many PPAR‐γ ligands were shown to inhibit tumor growth and progression in preclinical models of lung cancer, by modulating various cellular processes in cancer cells, stromal cells, and tumor microenvironment. However, some recent studies have also demonstrated the pro‐cancer effects of PPAR‐γ, suggesting that the function of PPAR‐γ in cancer may be related to cancer type and stage.^39^, ^60^ These data imply the possibility that ADAR1 contributes to the poor prognosis of LUAD and the suppressive TME through the PPAR signaling pathway. However, this still needs to be confirmed by more in‐depth studies.

In summary, LUAD highly expressing ADAR1 tends to metastasize to lymph nodes and has a suppressive TME, and patients with high ADAR1 expression have poorer OS and DFS than those with low ADAR1 expression. However, there were also some limitations in the present study. First, the results may be biased due to the limited sample size. Second, the correlation between ADAR1 and LNM needs to be confirmed with more effort to unveil the underlying mechanism. Then, the specific mechanism by which ADAR1 regulates the infiltration of diverse immune cells in LUAD requires further exploration, and this is an undergoing work of us. Given the role of the tumor microenvironment in tumor therapy, particularly in immunotherapy, our study will provide a basis for a further understanding of the regulatory relationship between tumor cells and the immune microenvironment in LUAD.

## AUTHOR CONTRIBUTIONS

**Wendi Yang:** Data curation (lead); formal analysis (lead); investigation (lead); methodology (lead); validation (lead); writing – original draft (lead). **Kehong Chen:** Formal analysis (equal); investigation (equal); methodology (equal). **Qian Yu:** Data curation (equal); methodology (equal). **Rongxin Liao:** Investigation (equal). **Hengqiu He:** Investigation (equal); validation (equal). **Yuan Peng:** Supervision (equal); writing – review and editing (equal). **Zhenzhou Yang:** Conceptualization (equal); funding acquisition (equal); writing – review and editing (equal). **Xiaoyue Zhang:** Conceptualization (equal); funding acquisition (equal); project administration (lead); supervision (equal); writing – review and editing (equal).

## CONFLICT OF INTEREST STATEMENT

All authors declare that they have no competing interests or financial conflicts to disclose.

## ETHICS STATEMENT

All procedures performed in studies involving human participants were in accordance with the ethical standards of the institutional and/or national research committee and with the 1964 Helsinki Declaration and its later amendments or comparable ethical standards. Informed consent was obtained from all individual participants involved in the study.

## Supporting information


Figure S1.

Figure S2.

Figure S3.

Figure S4.

Table S1.

## Data Availability

The authors confirm that the data supporting the findings of this study are available within the article.
