# Supplementary figures and images for: ADAR1 is a prognostic biomarker and is correlated with immune infiltration in lung adenocarcinoma
Source: Cancer Med. 2023 May 10;12(13):14820–32. doi: 10.1002/cam4.6044 (PMC10358204; doi:10.1002/cam4.6044)

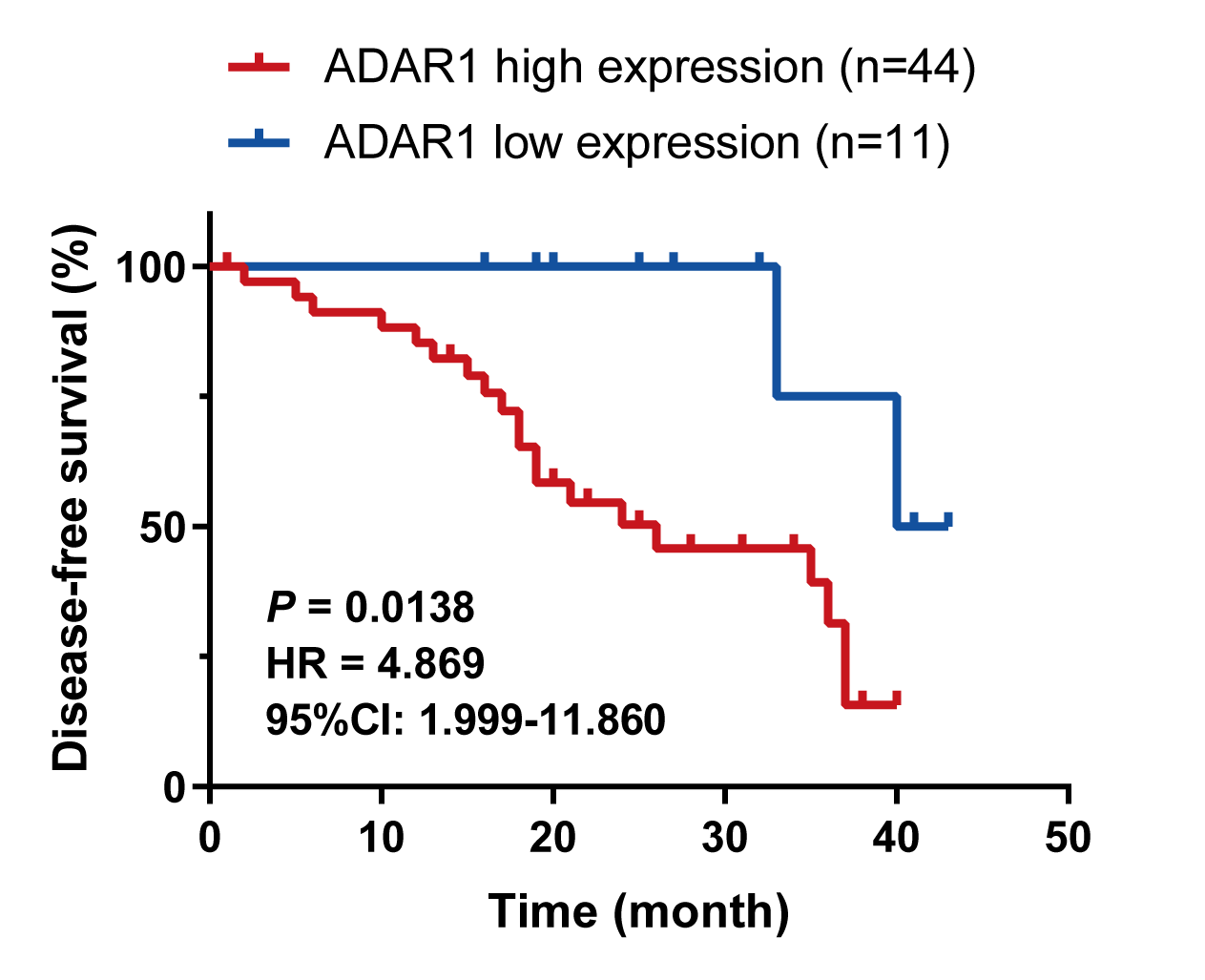

Supplement: Supplementary file 1 — Figure S1. [file CAM4-12-14820-s001.tif]

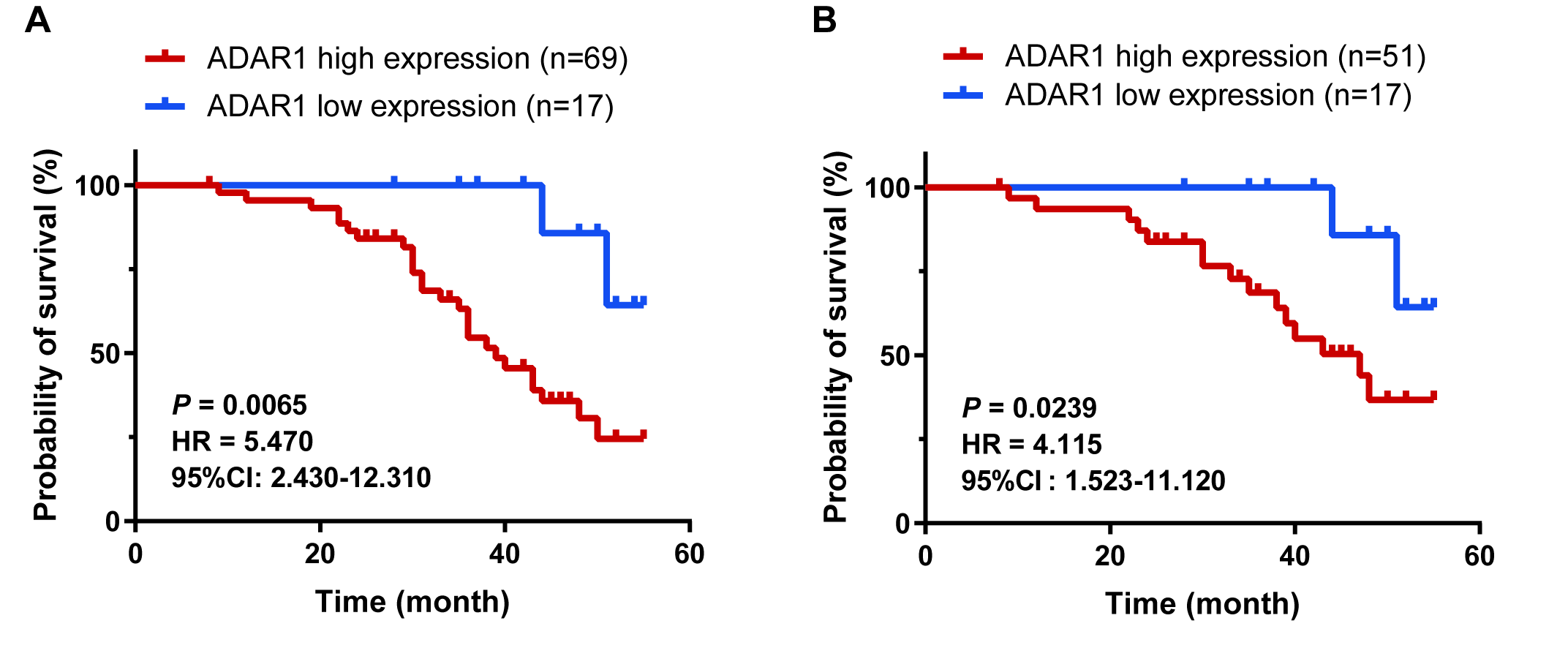

Supplement: Supplementary file 2 — Figure S2. [file CAM4-12-14820-s005.tif]

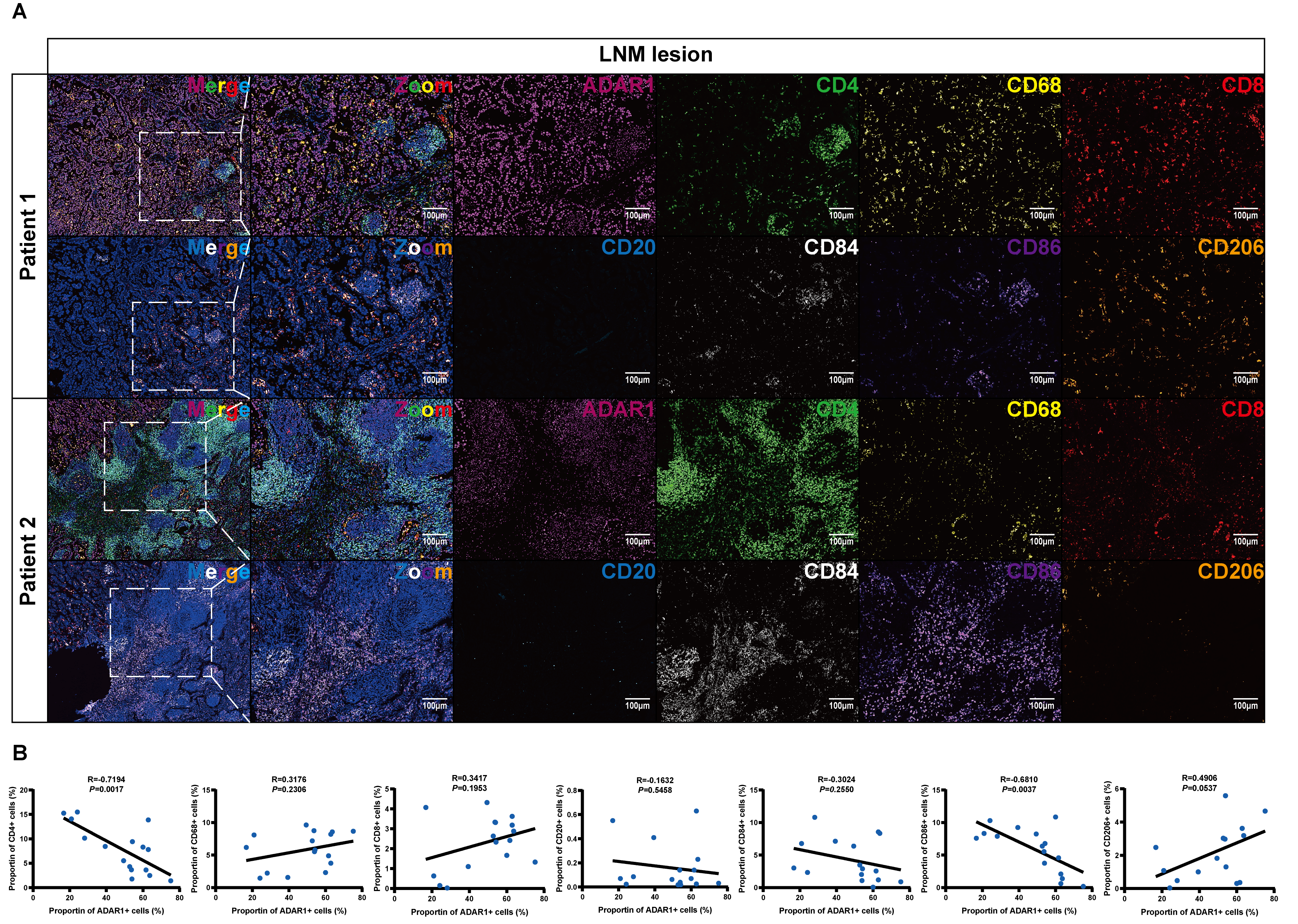

Supplement: Supplementary file 3 — Figure S3. [file CAM4-12-14820-s004.tif]

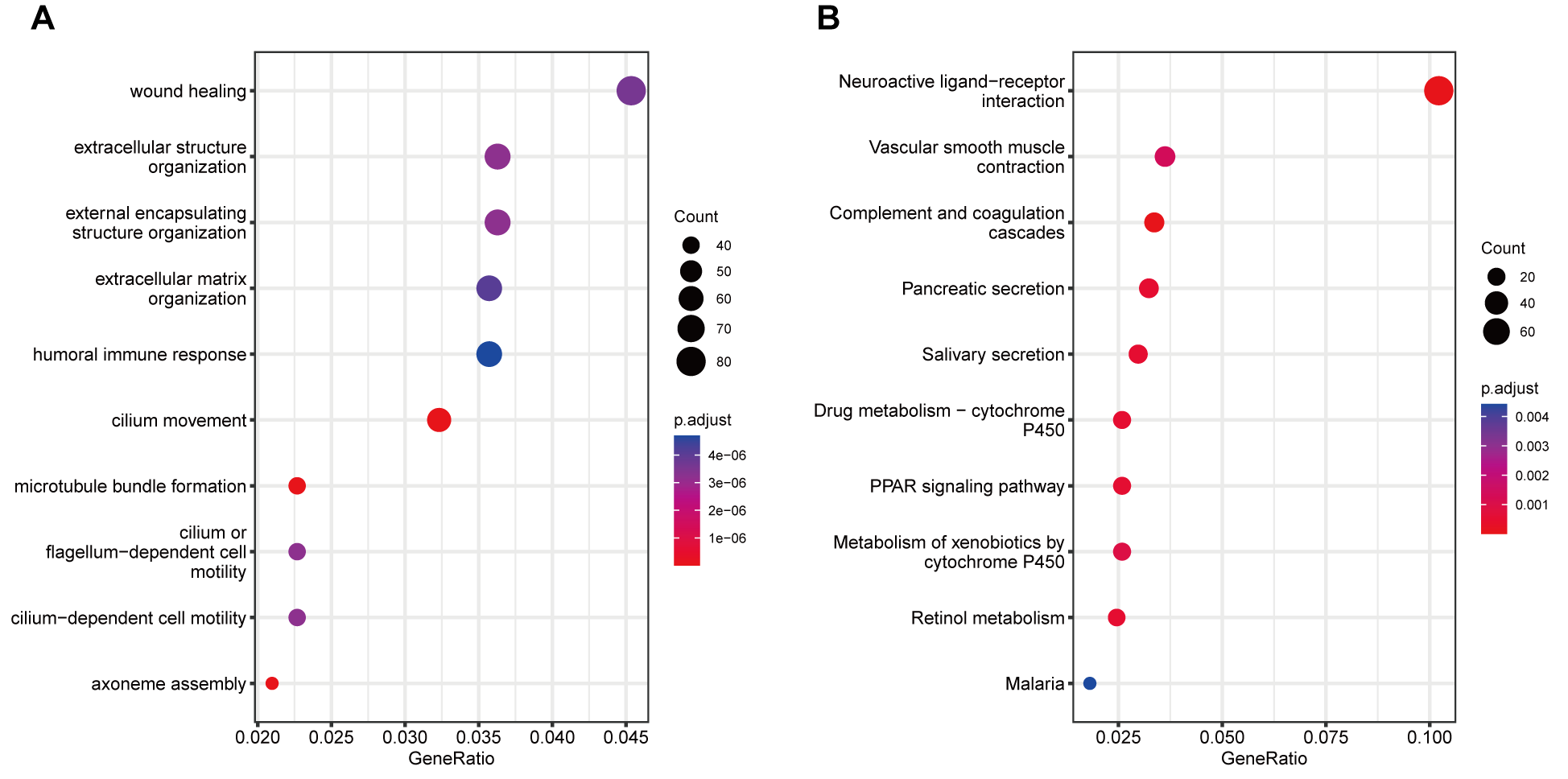

Supplement: Supplementary file 4 — Figure S4. [file CAM4-12-14820-s003.tif]
