# Supplementary material for: ADAR1 is a prognostic biomarker and is correlated with immune infiltration in lung adenocarcinoma
Source: Cancer Med. 2023 May 10;12(13):14820–32. doi: 10.1002/cam4.6044 (PMC10358204; doi:10.1002/cam4.6044)
Supplement: Supplementary file 5 — Table S1. [file CAM4-12-14820-s002.docx]

**Supplementary Materials**

**Figure legends**

**Figure S1. Kaplan-Meier curves of disease-free survival (DFS) of LUAD patients.**

K-M survival analysis of DFS for LUAD patients according to the level of ADAR1 protein expression. (* *P* < 0.05).

**Figure S2. The effect of ADAR1 expression on the prognosis of LUAD patients.**

**(A)** The effect of ADAR1 expression on overall survival (OS) of patients without lymph node metastasis. **(B)** The effect of ADAR1 expression on OS of patients with clinical stage Ⅰ and Ⅱ. (***P* < 0.01, * *P* < 0.05).

**Figure S3.** **Relationship of ADAR1 protein and immune cell infiltration in lymph node metastasis tissues.**

**(A)** Representative images of multiplex IHC staining of lymph node metastasis tissues. Immune panel: ADAR1+ (magenta), CD4^+^ T cells (CD4+, green), pan-macrophages (CD68+, yellow), CD8^+^ T cells (CD8+, red), B cells (CD20+, celeste), MDSCs (CD84+, white), M1 macrophages (CD86+, purple), and M2 macrophages (CD206+, orange). **(B)** Spearman’s correlation analysis between ADAR1+ tumor cell density and the density of various infiltrated immune cells in lymph node metastasis. R, Spearman coefficient.

**Figure S4.** **GO and KEGG enrichment analysis of the** **up-regulated genes.**

**(A)** Top significantly enriched GO terms of upregulated differentially expressed genes (DEGs). The x-axis represents the gene ratio, and the y-axis the significantly enriched GO terms. The depth of the color represents the adjusted *P*-value. The area of circle in the graph means gene counts. **(B)** Significantly enriched activated KEGG pathways. The vertical items are the names of KEGG terms, and the length of horizontal graph represents the gene ratio. The depth of the color represents the adjusted *P*-value. The area of circle in the graph means gene counts.

**Table S1. Relationship between ADAR1 and** **clinicopathological features in LUAD**

| Characteristics | n | ADAR1 expression | | χ^2^ | P-value |
| --- | --- | --- | --- | --- | --- |
|  |  | High | Low |  |  |
| Age |  |  |  |  |  |
| < 60  ≥ 60 | 44  56 | 35  48 | 9  8 | 0.665 | 0.415 |
| Gender |  |  |  |  |  |
| Male  Female | 49  51 | 41  42 | 8  9 | 0.031 | 0.861 |
| Lymph node metastasis | | |  |  |  |
| Negative  Positive | 68  32 | 51  32 | 17  0 | 9.639 | **0.002** |
| T stage |  |  |  |  |  |
| T1  T2  T3 | 47  29  16 | 34  26  15 | 13  3  1 | 7.644 | 0.054 |
| T4 | 8 | 8 | 0 |  |  |
| N stage |  |  |  |  |  |
| N0  N1  N2 | 68  26  6 | 51  26  6 | 17  0  0 | 9.639 | **0.008** |
| M stage |  |  |  |  |  |
| M0  M1 | 96  4 | 79  4 | 17  0 | 0.853 | 0.356 |
| Clinical staging |  |  |  |  |  |
| Ⅰ  Ⅱ  Ⅲ  Ⅳ | 64  22  9  5 | 48  21  9  5 | 16  1  0  0 | 8.189 | **0.0423** |
